# Initial Experience of Robot-Assisted Nephroureterectomy without Intraoperative Repositioning Using a New Robotic Surgical System (KD-SR-01TM)

**DOI:** 10.1155/2024/2466828

**Published:** 2024-08-10

**Authors:** Jie Dong, Weifeng Xu, Zhigang Ji

**Affiliations:** Department of Urology Peking Union Medical College Hospital Peking Union Medical College Chinese Academy of Medical Sciences, Beijing, China

## Abstract

**Background:**

Robot-assisted nephroureterectomy (RANU) has been more and more applied since 21st century. However, the high cost limits the widespread use of robot system. A relatively low-cost new robotic surgical system (KD-SR-01™) has recently been developed in China.

**Objective:**

To assess the safety and efficacy of the KD-SR-01™ Surgical System in RANU.

**Methods:**

Patients with upper-tract urothelial tumor and undergoing RANU with the KD-SR-01™ Robotic System were prospectively included. Surgeries were all performed by a single surgeon. Patients' demographic and clinical characteristics, perioperative data, pathology findings, and follow-up data were collected. *Key Findings*. 9 patients were enrolled in this study, and the surgeries went smoothly with no conversion to open. The 1^st^ docking time, the 2^nd^ docking time, and the operation time were 222 s, 169 s, and 202 min respectively. No equipment-related adverse events occurred. All patients were followed up for at least 3 months, and one patient experienced bladder recurrences. *Conclusions and Clinical Implications*. This study is the first to verify that the KD-SR-01™ robot system is effective and safe in RANU and has advantages in terms of its rotation boom during redocking and its price. This trial is registered with ChiCTR2200056672.

## 1. Introduction

Upper-tract urothelial carcinoma (UTUC) is a relatively uncommon disease, accounting for 5% of all urothelial tumors [1]. The gold standard of management of high-risk UTUC has been radical nephroureterectomy (NU) and excision of the bladder cuff [2]. Since NU needs to operate in the abdomen and pelvis concurrently, a long incision is required during open surgery. Laparoscopic NU has replaced the conventional open procedure and is accepted as the preferred surgical approach for the treatment of UTUC.

In 2006, Rose first reported a robot-assisted nephroureterectomy (RANU) using the da Vinci® system (Intuitive Surgical, Inc., Mountain View, CA, USA) [3]. Since then, many studies have suggested the safety and advantages of RANU [4, 5], and robotic surgery has become synonymous with Da Vinci®'s robot. Compared with traditional laparoscopic surgery, the Da Vinci® robotic system offers certain advantages such as three-dimensional optics, magnified view with depth perception, fully articulating instruments, tremors filtering, and a comfortably seated position for the surgeon. These advantages lead to better peri- and postoperative outcomes in the RANU procedure [6].

However, the high cost of Da Vinci® robot limits the development and application of this technology in developing countries. Herein, we introduce a new endoscopic surgery system, KD-SR-01™ (SuZhou Kang Duo Robot Co., 86 Ltd., Suzhou, China), which is independently developed by China, with independent intellectual property rights, and has recently been approved for registration (National Medical Products 131 Administration, NMPA) of CHINA. A prospective, single-center study was conducted to verify the effectiveness and safety of this KD-SR-01™ Robot System in RANU.

## 2. Patients and Methods

### 2.1. Patients

Patients presenting in Peking Union Medical Collage Hospital from October 2022 to July 2023 with a diagnosis of upper-tract urothelial tumor and undergoing RANU using the KD-SR-01™ robot system were included in this prospective, single-center study. The inclusion criteria included 18∼75 years old, clinically localized disease with no previous abdominal surgery or other uncorrected systemic diseases, willing to sign the informed consent form, and to have follow-up visits for at least 3 months. This study was approved by the ethics committee of our institution (approval no.: ks2022481).

### 2.2. Surgeon

All of these RANUs were carried out by a single surgeon (male) and a single assistant (female). This surgical team had adequate experience of laparoscopic nephroureterectomy and had accomplished da Vinci® procedures for more than 500 cases as well as KD-SR-01™ robotic surgeries for over 80 cases.

### 2.3. KD-SR-01™ Robot System

**Figure 1:**
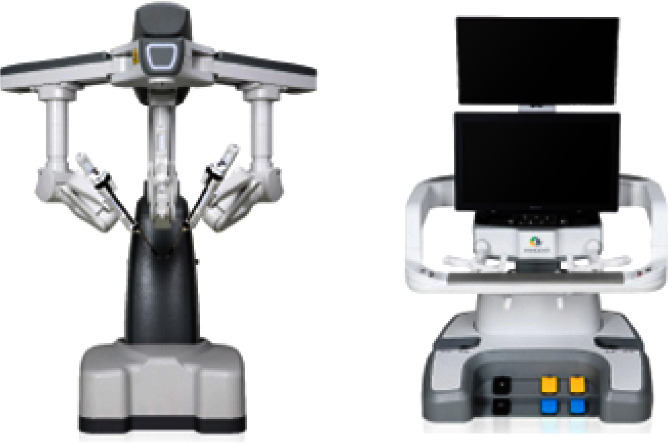
KD-SR-01™ robot endoscopic surgery system.

The KD-SR-01™ robot endoscopic surgery system is composed of an operating arm system, a doctor's console, and an imaging system (Figure 1).

The operating arm system is the operating part of the endoscopic surgery system, including 2 instrument arms and 1 laparoscopic arm, which provide support for surgical instruments and laparoscopic equipment beside the patient's bed during surgery. The surgical instrument has 7 degrees of freedom of movement, which can filter the tremor of the hand. The surgical assistant works next to the operating arm system to assist the surgeon in changing the surgical instruments and adjusting the laparoscope.

The doctor's console is the main part of the endoscopic surgery system. Unlike the da Vinci®, the console of the KD-SR-01™ robot system is an open platform. The surgeon only needs to sit in front of the doctor's control platform during surgery and observe the intraoperative images through the monitor. The surgeons can get 3D images from the monitor by simply wearing polarized glasses without bending his neck forward. Through the control of two main hand manipulators and foot pedal devices, the surgical instruments are controlled. The surgical arm and surgical instruments simulate the doctor's hand manipulator and perform the operation.

### 2.4. Operation

#### 2.4.1. Patient Position

After general anesthesia induction, the patient was placed in a 70° contralateral decubitus position with desk flexion and a slightly Trendelenburg tilt.

#### 2.4.2. Abdominal Surgery Phase

**Figure 2:**
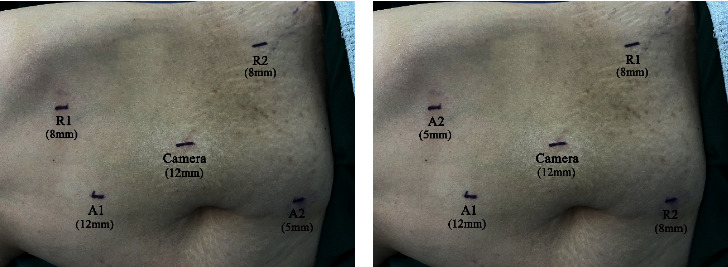
Port placement for left-side robot-assisted nephroureterectomy. (a) Port placement for the abdominal surgery phase. (b) Port placement for the pelvic surgery phase. R1: robot arm 1; R2: robot arm 2; A1: auxiliary cannula 1; A2: auxiliary cannula 2.

Veress needle was placed on the affected side 5 cm away from the umbilicus, at the outer edge of the rectus abdominis, and pneumoperitoneum was established. A 12-mm trocar was inserted, and a 30° 3D video laparoscope was placed. 8-mm robotic trocar was placed 5 cm below the costal margin of the middle clavicle line and 5 cm above the anterior superior iliac spine. Monopolar scissors and bipolar forceps were inserted after docking the robotic arms. 5-mm and 12-mm cannulas were placed midline 5 cm above and below the umbilicus for assistance (Figure 2(a)). After separating the adhesion, we mobilized the colon, cut the colonic ligament, and turned the colon to the ventral side. The renal artery and vein were dissected, clamped by Hem-o-lok clips, and cut separately. The kidney was then isolated. We dissected the ureter downwards to the level of the iliac vessels. Then, we removed the robotic surgical instruments and released the cannulas.

#### 2.4.3. Pelvic Surgery Phase

We rotated the boom of robotic arms to the pelvic part. The video laparoscope still used the cannula 5 cm lateral to the umbilicus. We swapped the auxiliary cannula 5 cm below the umbilicus with the robotic trocar below the costal margin at the midline of the clavicle line (Figure 2(b)). Monopolar scissors and bipolar forceps were inserted again after docking the robotic arms. We continued dissecting the ureter downwards to the bladder until the distal ureter was excised with the bladder cuff. We removed the monopolar scissors with a needle driver and closed the bladder defect by two-layer running sutures. Lymphadenectomy was performed at either abdominal surgery phase or pelvic surgery phase. Finally, we placed a draining tube, extended A1 incision along the midline to remove the specimen, and closed the skin incisions.

### 2.5. Study Variables

Patient age, sex, body mass index (BMI), tumor size, tumor location, conversion during surgery, docking time (DT), operative time (OT), estimated blood loss (EBL), perioperative transfusion, perioperative complication, equipment-related adverse events, postoperative hospitalization time, 30-day readmission rate, pathological tumor stage, positive margin rate, and follow-up data were collected and analyzed. Tumor size and location were assessed by preoperative abdominal enhanced CT. The first docking time (1^st^ DT) was defined as the time from moving the surgical system to the operating table until the last cannula was first docked to the corresponding surgical arm. The second docking time (2^nd^ DT) was defined as the time between the cessation of the robotic arm operation during the abdominal surgery phase and the start of the robotic arm operation during the pelvic surgery phase. Operative time was from skin incision to completion of incision suture. Continuous variables were described as the median [interquartile range (IQR), p25–p75].

## 3. Results

### 3.1. Demographics and Tumor Characteristics

From October 2022 to July 2023, 9 patients met the inclusion and were enrolled in our study. The baseline demographics and clinical characteristics are summarized in Table 1.

### 3.2. Perioperative Data

As shown in Table 2, all RANU were performed successfully with no conversion to open. The 1^st^ docking time, the 2^nd^ docking time, and the operation time were 216 s (187 s, 231 s), 164 s (130 s, 200 s), and 196 min (189 min, 207 min), respectively. All of these patients had minimal intraoperative bleeding (100 ml) and did not require blood transfusion.

There were no intraoperative complications. One patient had a preoperative CT scan indicating suspicious lymph node metastasis near the left iliac vessels. Pelvic lymph node dissection was performed during the surgery, and a lymphorrhagia appeared postoperatively. The other patient experienced postoperative fever. They both improved on their own within 3 days. There were no equipment-related adverse events occurred. All enrolled patients recovered well and discharged shortly. There was no readmission within 30 days.

### 3.3. Pathology Findings and Follow-Up Data

The pathological results (Table 3) showed that 6 patients had pT2 stage diseases, 3 patients belonged to pT3 stage, and all tumors were high-grade urothelial carcinoma. All patients had negative margins. One patient was confirmed to have lymph node metastasis by postoperative pathology.

All patients were followed up for at least 3 months. The average follow-up time was 6 months (4 months, 8 months) (Table 3). For patients with T3 stage diseases, adjuvant chemotherapy was recommended. A patient with lymph node metastasis was diagnosed with a bladder tumor 3 months after surgery and underwent transurethral resection of bladder tumors (TURBT). The surgery went smoothly, and the patient was discharged quickly after surgery.

## 4. Discussion

Upper-tract urothelial carcinoma is a relatively rare disease in urology. In recent years, minimally invasive nephroureterectomy, such as laparoscopic and robot-assisted surgery, has become the preferred approach used in the surgical treatment of UTUC [5]. Robot-assisted nephroureterectomy has been more and more applied in clinical practice since 21st century [7]. In this study, we first reported the application of domestic robotic system, the KD-SR-01™ Robot System, in RANU. All tumors were completely removed, and all patients successfully completed the surgery without conversion to open surgery. The safety and effectiveness of the system in clinical application are preliminarily verified.

For nephroureterectomy, the main difficulty lies in the pelvic surgery phase. Due to the deep location, it is difficult to expose the end of the ureter and excise the bladder cuff during open surgery, while suturing the bladder is challenging for laparoscopic surgery. These issues are more obvious for overweight patients. Due to the flexibility of robot equipment, these difficulties can be easily handled in robot surgeries. In this study, the average BMI of the patients was 25.5, which exceeded the overweight level (BMI >25) according to the international standards [8]. Among them, one patient had a BMI of 30.1, reaching the level of obesity [8]. With the help of the KD-SR-01™ Robot System, the surgeries progressed smoothly and did not encounter any difficulties. This can once again verify the flexibility and effectiveness of this robot system.

During the same period, this surgical team performed 8 RANUs using the da Vinci robot system. When compared the perioperative outcomes of our study with those using da Vinci, we found no difference between two robotic systems (1^st^ docking time 216 s vs 230 s, operation time 196 min vs 200 min, intraoperative bleeding 100 ml vs 131 ml). It was interesting to find a shorter 2^nd^ docking time for the KD-SR-01™ Robot System than da Vinci® Si (164 s vs 448 s). This can be attributed to the liftable and rotatable suspended operating arm structure platform of the patient cart in the KD-SR-01™ Robot System, similar to da Vinci® Xi, which can avoid moving the position of patient cart and minimize the 2^nd^ docking time as much as possible. This demonstrated the convenience of the KD-SR-01™ Robot System.

Compared with traditional laparoscopic surgery, da Vinci® robot surgery provides a comfortable posture for surgeons, reduces the injury of waist and knee caused by surgery, and significantly reduces surgical fatigue [9]. However, Giberti [10] pointed out that 41.2% of surgeons reported a recurrent musculoskeletal disorder, mainly neck pain, which started from the beginning of the robotic experience. These aspects could be due to the fixed position of the console binocular viewer which could have produced an inadequate spinal posture with consequent musculoskeletal disorders. In contrast, the KD-SR-01™ Robot System provides an open console, and the surgeon can obtain a 3D image by simply wearing polarized glasses. This ensures the natural posture of the neck and is more ergonomic.

Despite the above advantages, the cost associated with the use of robotic systems may explain the fact that laparoscopic nephroureterectomy is still more frequently used worldwide. Bodner [11] estimated that the cost of robotic surgery was 1.5 times higher or more than that of traditional laparoscopic surgery. This makes it impossible for many medical institutions to perform robotic surgeries. However, for the KD-SR-01™ Robot System, this issue has been carefully considered and sufficiently eliminated. First, the cost of the robotic platform and disposable instruments for the KD-SR-01™ Robot System is much lower than da Vinci® robot surgery. Second, this three-arm system uses one less robotic instrument than a four-arm system, which can further reduce the cost of consumables in surgery. In this study, we have demonstrated that using three robotic arms and two assistant ports for RANU is safe and feasible. Third, the KD-SR-01™ Robot System can match the 3D laparoscopic display system of various brands, thereby reducing the purchase cost. All of these ensure that patients can receive robot-assisted surgeries at a lower and more acceptable price.

There are still some defects in this study. First, the number of patients included in the study is relatively small. Larger clinical studies are needed to provide more solid conclusions. Second, the follow-up time of the study patients is short, which is not enough to verify the long-term oncologic outcomes. Third, the surgical team participated in the study has very rich experience in both Da Vinci® and KD-SR-01™ robot systems, and the effectiveness and safety of this KD-SR-01™ robot system require more physicians to verify.

## 5. Conclusion

This article first reports the application of the KD-SR-01™ Robot System in robot-assisted nephroureterectomy. KD-SR-01™ is independently developed by China, with independent intellectual property rights, and has recently been approved for registration. Through this study, we verified the KD-SR-01™ Robot System's effectiveness and safety in RANU, and it has advantages in terms of its rotation boom during redocking and its price. The KD-SR-01™ Robot System, with the advantages of accuracy, dexterity, more ergonomic, and lower cost, is expecting clinical studies with more cases and longer follow-up time to further verify the conclusions of this experiment.

## Figures and Tables

**Table 1:** Demographics and clinical characteristics.

No	Sex	Age	BMI (kg/m^2^)	Tumor side	Tumor location	Tumor size (cm)
1	Male	65	30.1	Left	Renal pelvis	1
2	Female	62	22.8	Left	Ureter	5
3	Female	76	26.3	Right	Ureter	1.5
4	Female	70	25.4	Right	Ureter	4.4
5	Female	68	24.9	Right	Ureter	4.5
6	Male	71	22.8	Left	Renal pelvis	1
7	Male	51	25.1	Right	Ureter	3.7
8	Male	67	26.6	Left	Renal pelvis	5
9	Male	70	25.4	Right	Renal pelvis	3

**Table 2:** Perioperative data.

No	Conversion to open	1st docking time (s)	2nd docking time (s)	Operation time (min)	Estimated blood loss (ml)	Blood transfusion	Intraoperative complications	Postoperative complications		Equipment-related adverse events	Postoperative hospitalization time (d)	30-day readmission
								Clavien I-II	Clavien III-IV			
1	No	194	110	201	100	No	No	No	No	No	6	No
2	No	187	164	207	100	No	No	Lymphorrhagia	No	No	9	No
3	No	245	240	195	100	No	No	No	No	No	6	No
4	No	186	200	174	100	No	No	No	No	No	6	No
5	No	187	120	242	100	No	No	No	No	No	5	No
6	No	329	240	196	100	No	No	No	No	No	5	No
7	No	216	180	235	50	No	No	No	No	No	5	No
8	No	231	140	186	150	No	No	No	No	No	5	No
9	No	227	130	189	100	No	No	Fever	No	No	7	No

**Table 3:** Pathology findings and follow-up data.

No	Tumor stage	Tumor grade	Surgical margin	Lymph node stage	Follow-up (month)	Adjuvant therapy	Recurrence
1	2	High grade urothelial carcinoma	Negative	0	11	No	No
2	3	High grade urothelial carcinoma	Negative	1	9	Chemotherapy	Bladder tumor
3	2	High grade urothelial carcinoma	Negative	0	8	No	No
4	2	High grade urothelial carcinoma	Negative	0	7	No	No
5	2	High grade urothelial carcinoma	Negative	0	6	No	No
6	2	High grade urothelial carcinoma	Negative	0	5	No	No
7	3	High grade urothelial carcinoma	Negative	0	4	Chemotherapy	No
8	2	High grade urothelial carcinoma	Negative	0	4	No	No
9	3	High grade urothelial carcinoma	Negative	0	3	Chemotherapy	No

## Data Availability

The datasets generated during and/or analyzed during the present study are available from the corresponding author on reasonable request.
